# Supplementary material for: A general computational design strategy for stabilizing viral class I fusion proteins
Source: Nat Commun. 2024 Feb 13;15:1335. doi: 10.1038/s41467-024-45480-z (PMC10864359; doi:10.1038/s41467-024-45480-z)
Supplement: Supplementary file 1 — Supplementary Information [file 41467_2024_45480_MOESM1_ESM.pdf]

Supplementary Information for

A general computational design strategy for stabilizing viral class I fusion proteins

Karen J. Gonzalez, Jiachen Huang, Miria F. Criado, Avik Banerjee, Stephen M. Tompkins, Jarrod J. Mousa, Eva-Maria Strauch

*Corresponding author. Email: evas@wustl.edu

This PDF file includes:

Supplementary Figures 1 to 12

Supplementary Tables 1 to 7

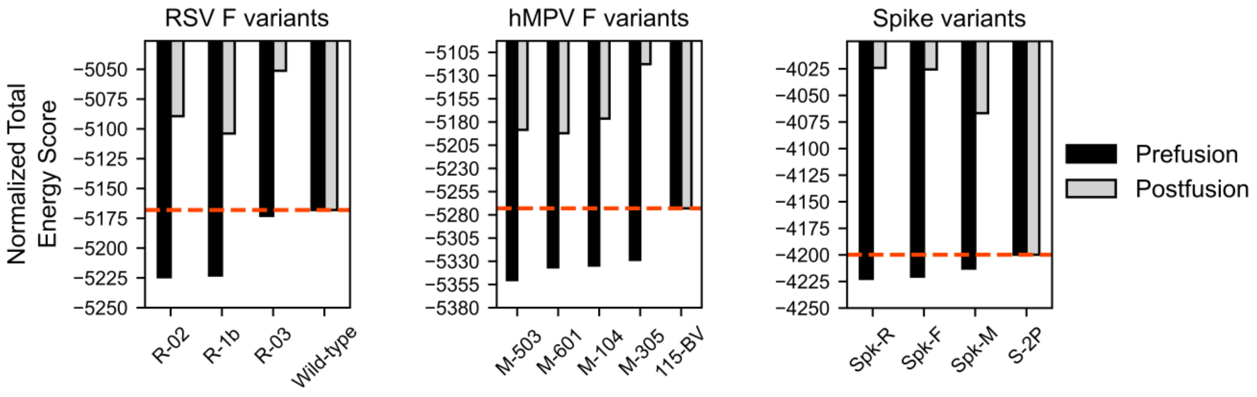

Supplementary Figure 1. Energy comparison between prefusion base constructs and designed variants. In black is represented the prefusion conformation while grey depicts the postfusion conformation. An orange dotted line highlights the normalized total energy of the starting sequences. To compare the energy gain or loss of each variant, all prefusion energies were normalized to the postfusion state using the ratio postfusion-energy base construct/prefusion-energy base construct. The energy gap between states is shown in Supplementary Table 1. Source data for all panels are provided as a Source Data file.

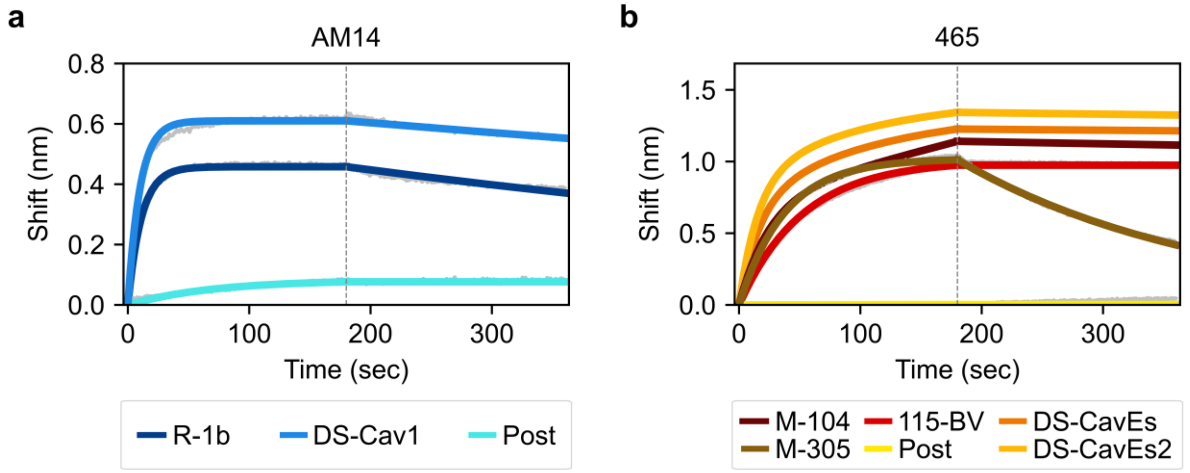

Supplementary Figure 2. Binding of designed variants and controls to prefusion-specific antibodies. (a) Binding of RSV F variants to antibody AM14. Binding constants are shown in Supplementary Table 2. “Post” stands for postfusion RSV F A2. **(b)** Binding of hMPV F variants to antibody 465. Binding constants are shown in Supplementary Table 3. “Post” stands for postfusion hMPV B2 F. In grey is shown the raw data, and in colors, the fitted curves. A dotted vertical line represents the end of the association time. Source data for all panels are provided as a Source Data file.

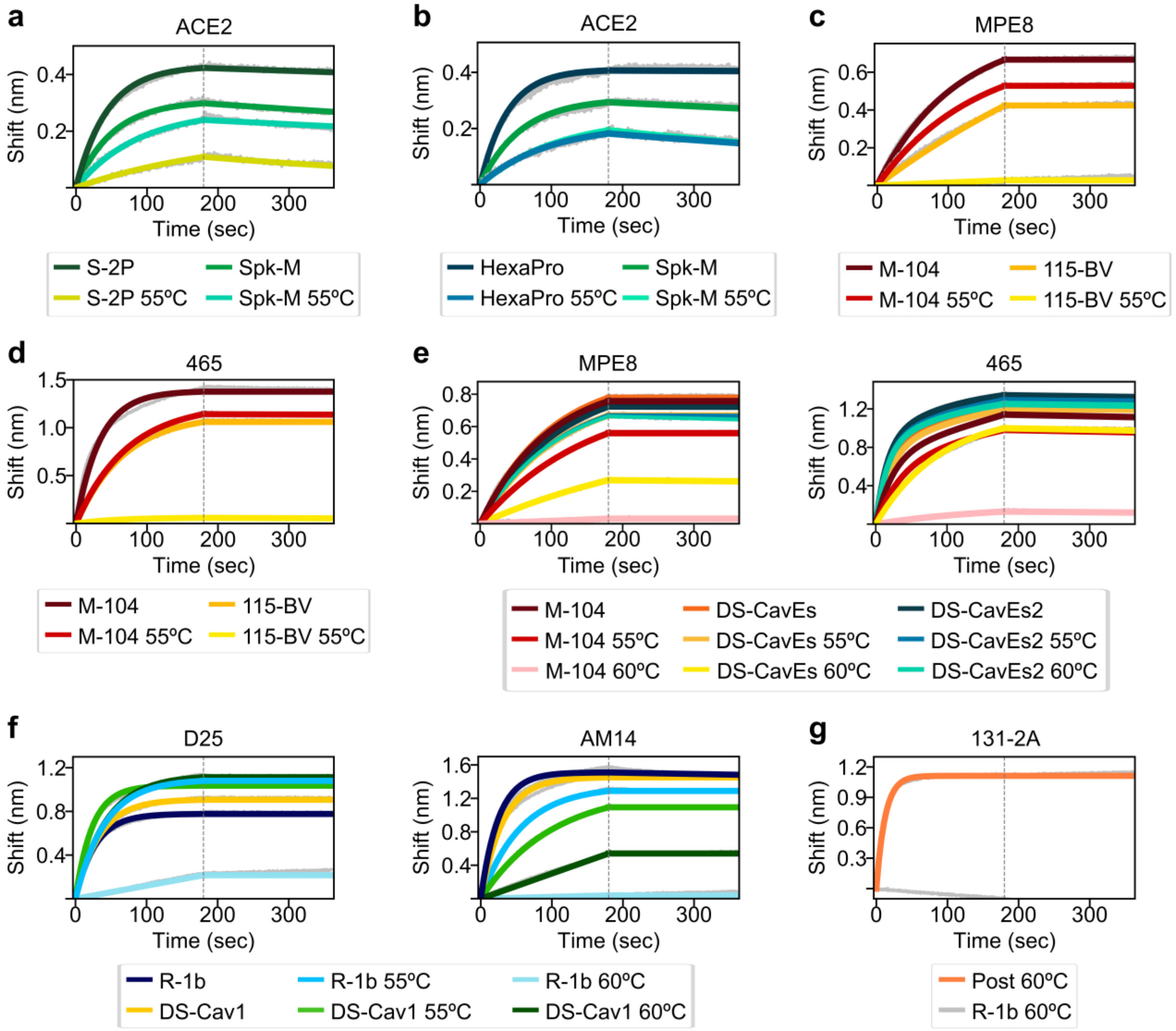

Supplementary Figure 3. Binding of designed variants and controls after heat treatment. (a) ACE2 binding to design Spk-M and the base construct S-2P. **(b)** ACE2 binding to design Spk-M and the next-generation immunogen HexaPro. **(c)- (d)** MPE8 and 465 binding to design M-104 and the base construct 115-BV. **(e)** MPE8 and 465 binding to design M-104 and next generation immunogens DS-CavEs and DS-CavEs2. **(f)** D25 and AM14 binding to design R-1b and the prefusion control DS-Cav1. **(g)** Binding of design R-1b and postfusion RSV A2 F ("Post") to the postfusion-specific antibody 131-2A after heating at 60°C. Since R-1b does not bind to 131-2A after heating, only the raw data is displayed for this protein. Binding constants are summarized in Supplementary Tables 2-4. In grey are shown raw data and in colors fitted curves. A dotted vertical line represents the end of the association time. Source data for all panels are provided as a Source Data file.

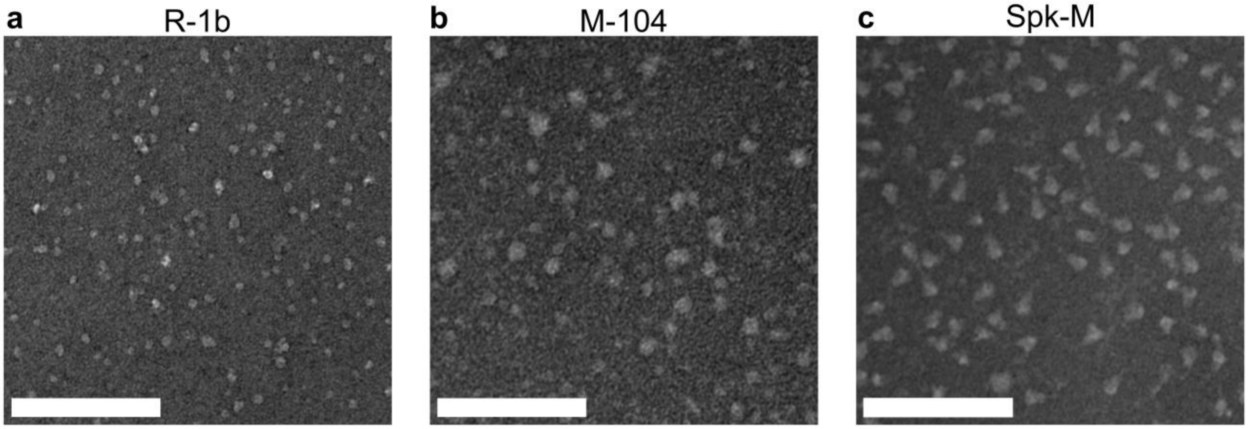

Supplementary Figure 4. Representative negative stain-electron microscopy of designs **(a)** R-1b, **(b)** M-104, and **(c)** Spk-M. Scale bar: 100 nm. A total of ten micrographs were collected from different areas of each grid.

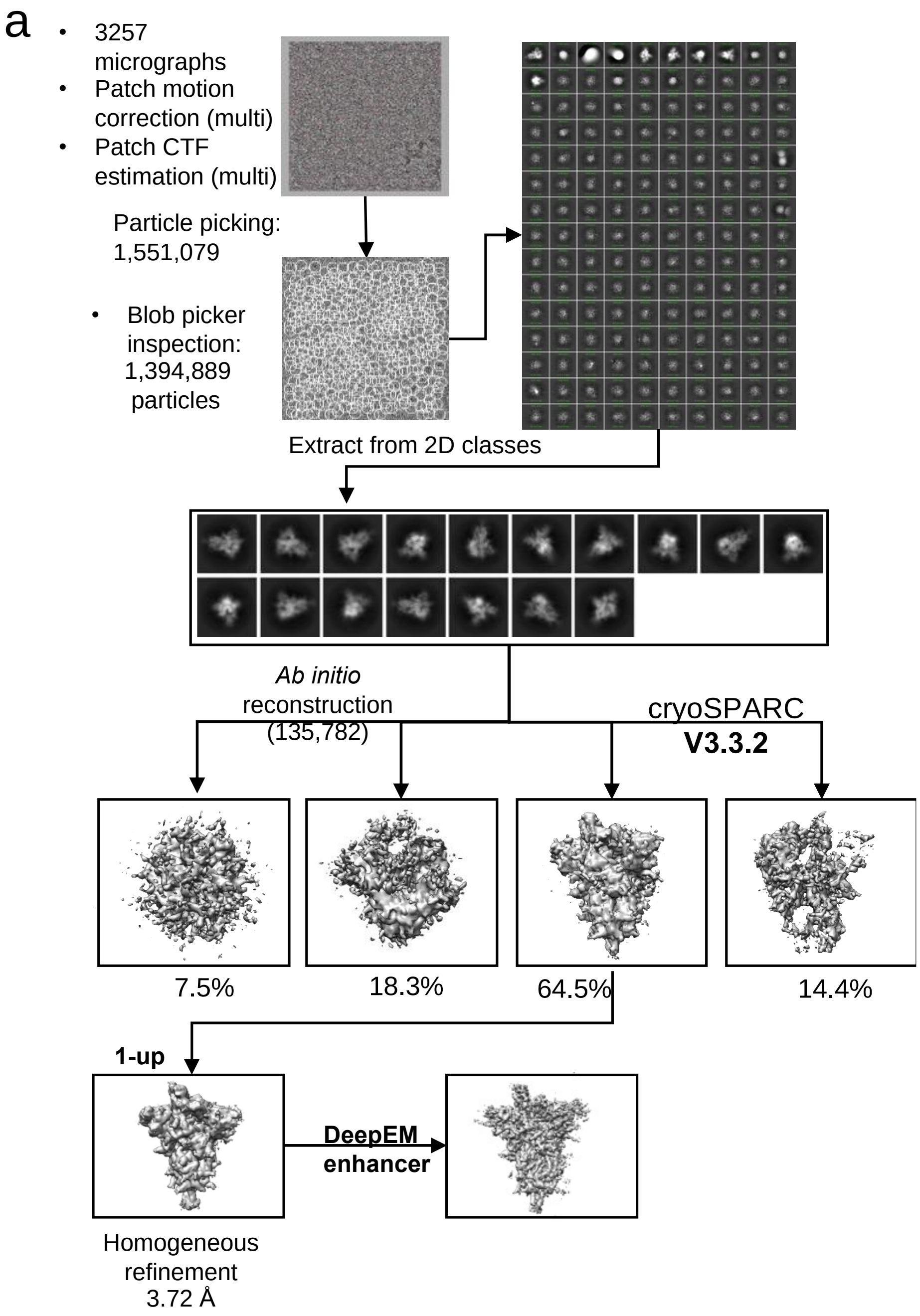
a
3257 micrographs
Patch motion correction (multi)
Patch CTF estimation (multi)
Particle picking: 1,551,079
Blob picker inspection: 1,394,889 particles
Extract from 2D classes
Ab initio reconstruction (135,782)
cryoSPARC V3.3.2
7.5%
18.3%
64.5%
14.4%
1-up
DeepEM enhancer
Homogeneous refinement 3.72 Å

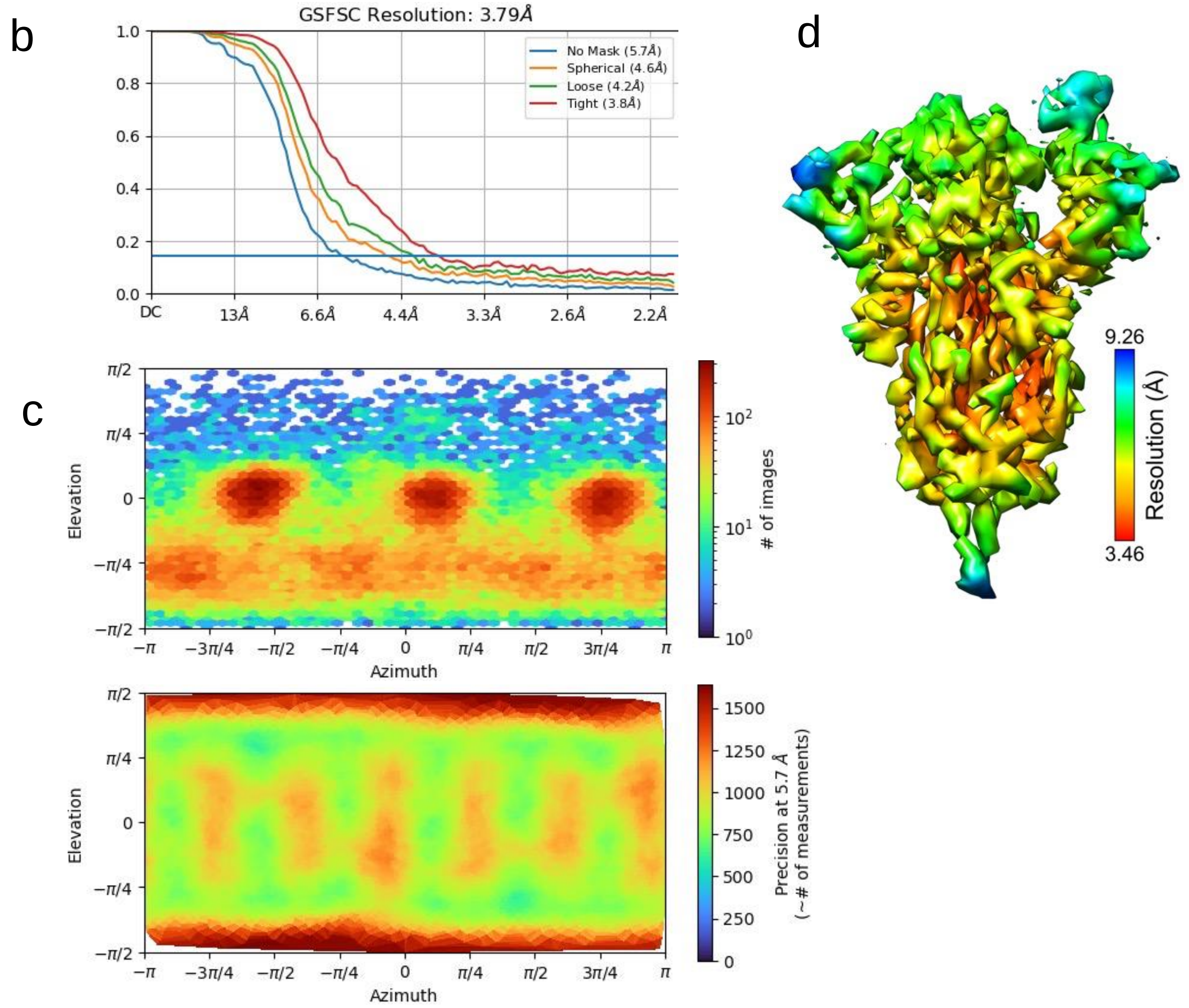

Supplementary Figure 5. Summary of cryo-electron microscopy data for design Spk-M. (a) Micrographs were processed in cryoSPARC V3.3.2 and final refinement in DeepEM enhancer. **(b)** FCS curve. **(c)** Direction Distribution and Posterior Precision Directional Distribution. **(d)** Local resolution map**.**

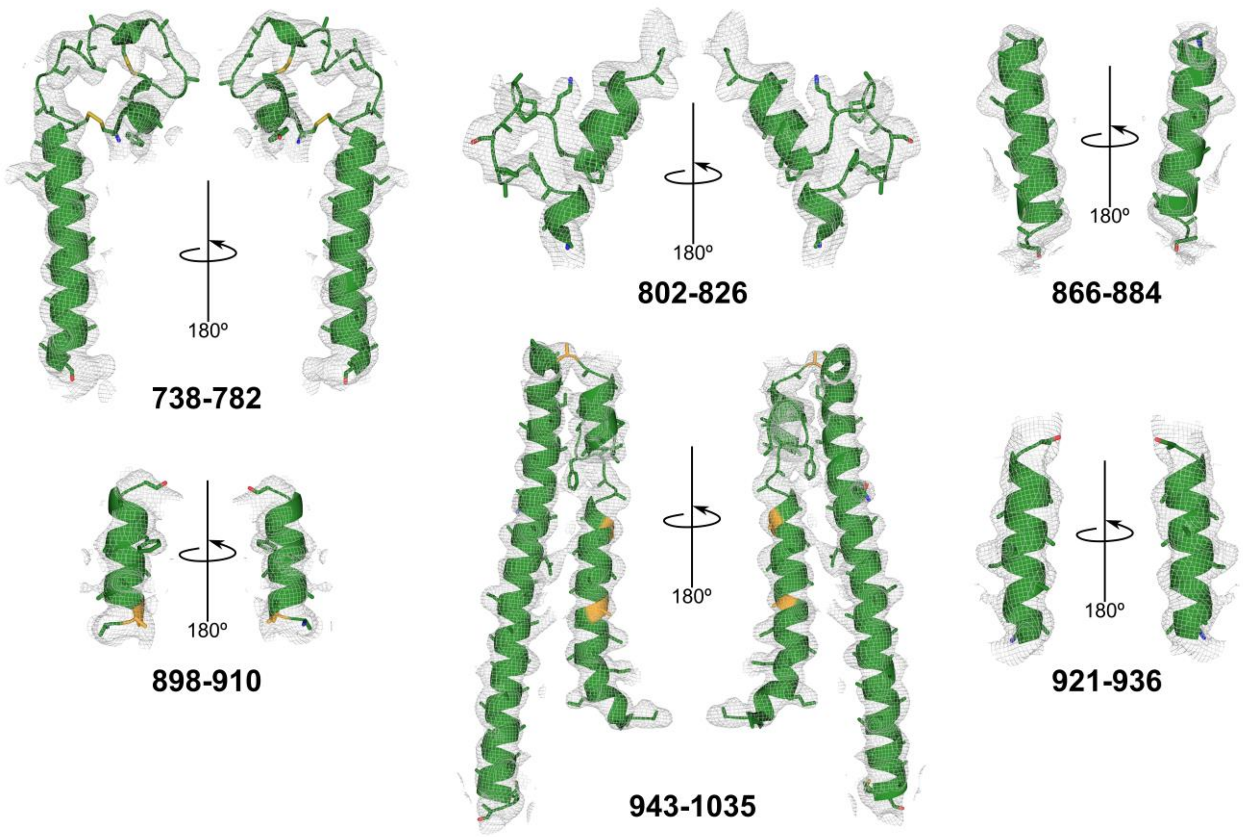

Supplementary Figure 6. Representative helical structures within the Spk-M S2 subunit fitted to cryo-EM densities. The Spk-M reconstruction model is shown in a combination of cartoon and sticks representation in green, with introduced mutations highlighted in yellow. Cryo-EM densities are depicted as a mesh representation in grey, contoured at a 3.5 level.

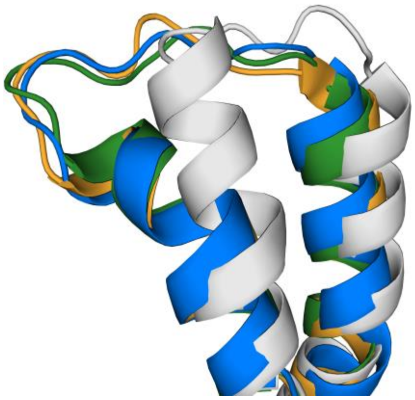

Supplementary Figure 7. Structural alignment between head residues of different RSV F variants. R-1b is displayed in blue and its parent construct PDB 5w23 is displayed in grey. In yellow and green are shown the DS-Cav1 structures PDB 4mmu and 5k6c, respectively. On display are residues 195-227.

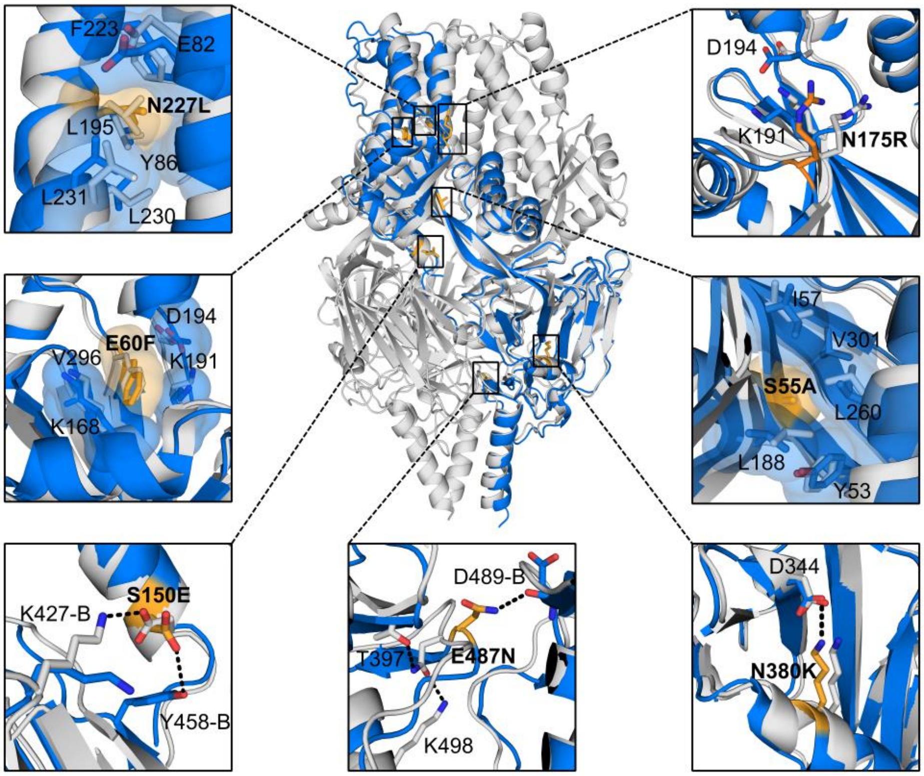

Supplementary Figure 8. Atomic interactions of all substitutions introduced in design R-1b compared to a computational model. The computational model of the protein is displayed as a trimeric structure in grey, while the crystal structure is displayed as a monomeric structure in blue, with introduced mutations in yellow. Each panel shows a magnified view of the atomic interactions involving each substitution (in yellow sticks), aligned with their computational model. Residues contributing to packing changes are displayed with translucent molecular surfaces, and black dotted lines represent hydrogen bonds or salt bridges.

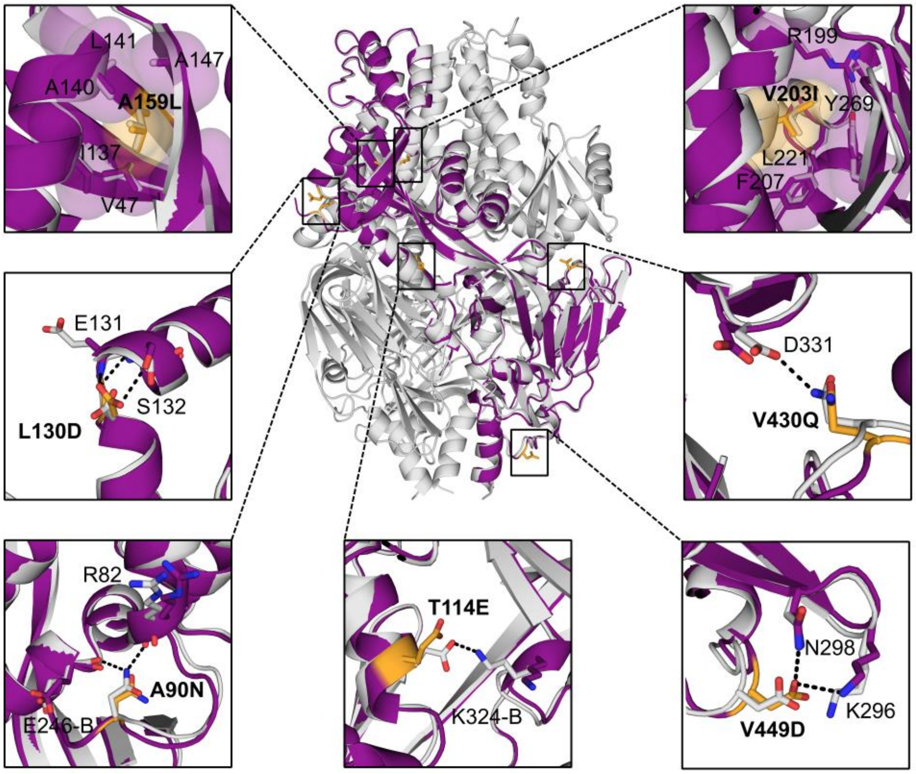

Supplementary Figure 9. Atomic interactions of all substitutions introduced in design M-104 compared to a computational model. The computational model of the protein is displayed as a trimeric structure in grey, while the crystal structure is displayed as a monomeric structure in purple with introduced mutations in yellow. Each panel shows a magnified view of the atomic interactions involving each substitution (in yellow sticks), aligned with their computational model. Residues contributing to packing changes are displayed with translucent molecular surfaces, and black dotted lines represent hydrogen bonds or salt bridges.

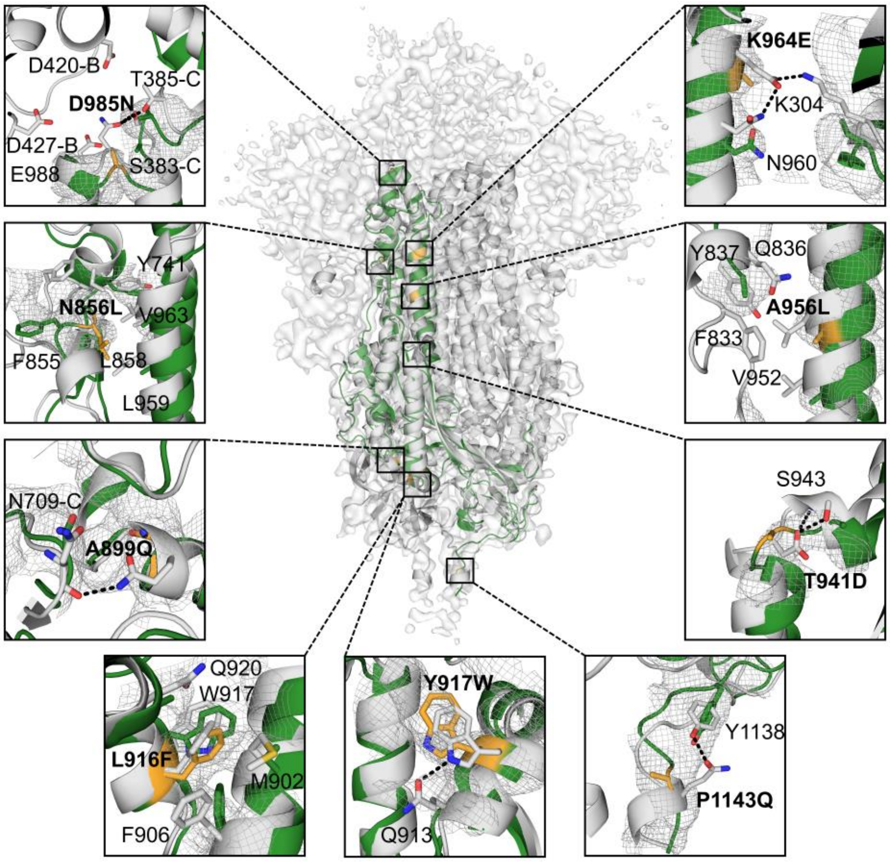

Supplementary Figure 10. Predicted atomic interactions of all designed substitutions introduced in the S2 subunit of design Spk-M. The computational model of the protein is displayed as a trimeric structure in grey, while the cryo-EM reconstruction model is displayed as a monomeric structure in green with introduced mutations in yellow. The Spk-M cryo-EM map is shown as a translucent surface in grey. Each panel shows a magnified view of the atomic interactions involving each substitution (in yellow sticks), aligned with their computational model. As density is missing in the overall map to assign the precise location of the side chains, we displayed existing density as a mesh representation to compare agreement with the computational model. Black dotted lines represent hydrogen bonds or salt bridges.

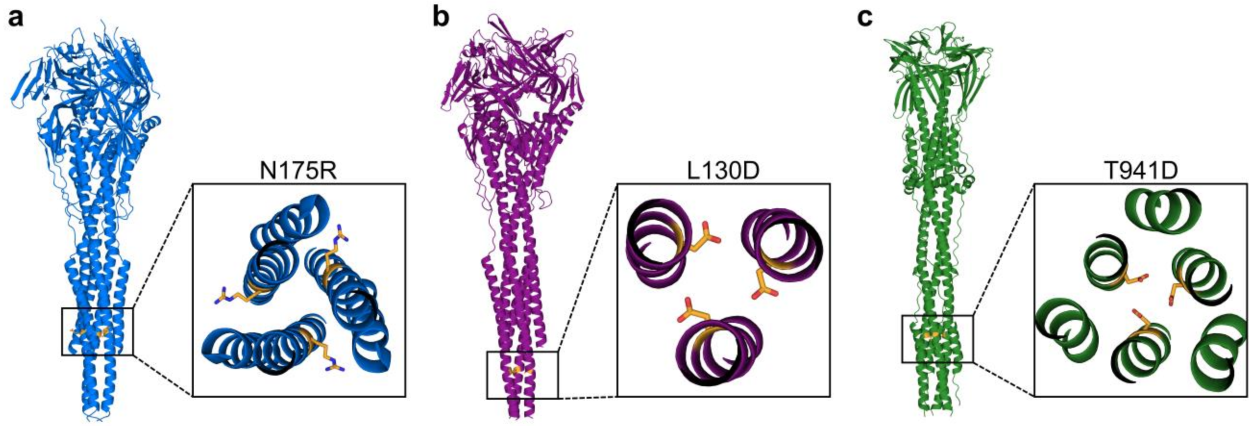

Supplementary Figure 11. Computational models of postfusion destabilizing substitutions introduced in (a) R-1b. **(b)** M-104. **(c)** Spk-M. Each panel shows a magnified view of the predicted rotamer configuration of each mutation. All substitutions are represented in yellow sticks.

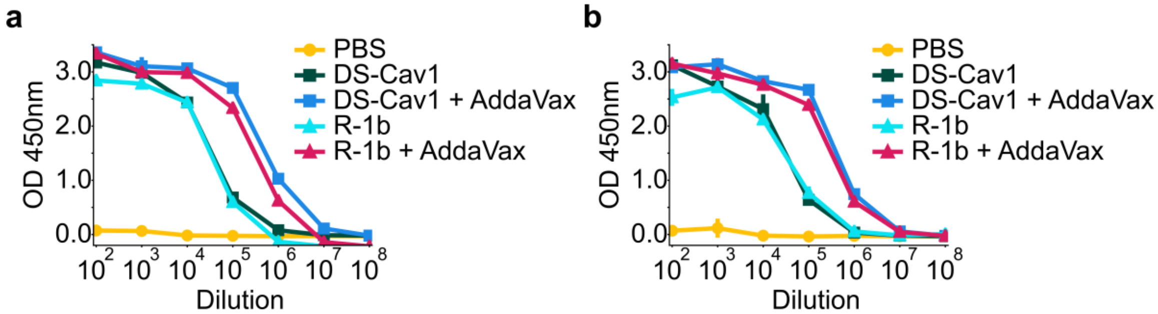

Supplementary Figure 12. Immunogenicity assessment of RSV F variants in mice using 2ug doses. (a) Serum RSV-specific IgG measured by ELISA three weeks post-boost. **(b)** Serum RSV-specific IgG measured by ELISA nine weeks post-boost. The markers on each line plot indicate mean values while the vertical lines represent the standard deviation. Values were calculated from three repetitions using pooled serum samples from mice within each immunization group (5 animals/group). Source data for all panels are provided as a Source Data file.

Supplementary Table 1. Comparison of normalized energy changes for each trimeric unit in pre- and postfusion states, stabilizing mutations, and thermal stability of designed fusion proteins.

Virus	Protein variants	***Normalized energy difference*** *Pre -vs- Postfusion*	**Stabilizing mutations**						Melting temperature (°C)
			**Cavity filling**	**Inter-protomer polar interactions**	**Intra-protomer polar interactions**	**Reduction of unsatisfied polars**	**Decrease charge repulsion**	**Postfusion destabilizing**	
**RSV**	Base construct (WT)	0*	N/A	N/A	N/A	N/A	N/A	N/A	N/A
	R-1b	119.1	E60F	S150E	N380K	S55A, N227L	E487N	N175R	62
	R-02	135.1	E60F, S150L	N/A	N380K	S55V, N227L	E487V	N175R	N/A
	R-03	121.7	E60F, S150L	N/A	N380K	S55L, N227F	E487V	N175R	N/A
**hMPV**	Base construct (115-BV)	0*	N/A	N/A	N/A	N/A	N/A	N/A	54.8
	M-104	158.2	A159L, V203I	A90N, T114E	V430Q, V449D	N/A	N/A	L130D	61.5
	M-305	210.7	A159I	A90N	G106R, G277D, A314K, V449D	N/A	E453P	L130D	56.7
	M-503	161.8	G106W, A107F, A159L, V162I, V203I	T114E	V430Q, V449D	N/A	N/A	L130D	N/A
	M-601	144.4	A159L, V191I	T114E, D209E, A216R	V430Q, V449D	S149T	N/A	L130D	N/A

**SARS-CoV-2**	Base construct (S-2P)	0*	N/A	N/A	N/A	N/A	N/A	N/A	45.7[a],62.5[b]
	Spk-M	146.1	L916F, Y917W, A956L	A899Q	K964E, P1143Q	N856L	D985N	T941D	61
	Spk-F	194.9	A956L, A1016I	E990R	G769K, P1143Q	N/A	N/A	T941D	61.5
	Spk-R	198.4	A1016I	E990R	G744T, G769K, N955D, P1143N	N/A	D985N	T941D	46.5[a], 60.3[b]

*The base construct's pre- and postfusion energies are normalized.
N/A = not applicable
a) First apparent melting temperature
b) Second apparent melting temperature

Supplementary Table 2. Binding kinetics of RSV F variants obtained by biolayer interferometry.

**Protein Variants**	**Assay temperature (°C)**	**Antibodies**								
		**D25**			**AM14**			**131-2A**		
		koff(1/s)	kon(1/Ms)	KD(M)	koff(1/s)	kon(1/Ms)	KD(M)	koff(1/s)	kon(1/Ms)	KD(M)
R-1b	RT	N/A	1.79E+05<br>1.51E+05<br>1.44E+05	N/A	1.24E-04<br>1.01E-04<br>1.11E-04	2.19E+05<br>2.03E+05<br>2.27E+05	5.68E-10<br>4.97E-10<br>4.89E-10	4.00E-04<br>3.10E-04<br>3.53E-04	3.37E+04<br>4.79E+04<br>4.75E+04	1.19E-08<br>6.47E-09<br>7.43E-09
	55	N/A	1.56E+05<br>1.20E+05<br>1.21E+05	N/A	N/A<br>1.15E-05<br>1.31E-05	8.87E+04<br>8.63E+04<br>9.91E+04	N/A<br>1.34E-10<br>1.33E-10	N/A	N/A	N/A
	60	N/A	92.7<br>83.8<br>54.6	N/A	N.B	N.B	N.B	N.B	N.B	N.B
DS-Cav1	RT	N/A	1.21E+05<br>1.87E+05<br>1.49E+05	N/A	N/A	1.61E+05<br>2.04E+05<br>1.81E+05	N/A	0.001<br>3.17E-04<br>3.07E-04	8.29E+03<br>9.19E+03<br>1.76E+04	1.33E-07<br>3.44E-08<br>1.74E-08
	55	N/A	2.13E+05<br>1.60E+05<br>1.42E+05	N/A	N/A	5.03E+04<br>5.85E+04<br>5.01E+04	N/A	N/A	N/A	N/A
	60	N/A	1.15E+05<br>1.35E+05<br>1.32E+05	N/A	N/A	3.02E+04<br>1.31E+04<br>5.39E+03	N/A	N/A	N/A	N/A

RSV A2 F (postfusion)	RT	N.B	N.B	N.B	N.B	N.B	N.B	N/A	4.00E+05<br>4.09E+05<br>4.85E+05	N/A
	60	N/A	N/A	N/A	N/A	N/A	N/A	N/A	5.22E+05<br>4.82E+05<br>4.52E+05	N/A

RT= room temperature
N.B = no binding
N/A = not applicable

Supplementary Table 3. Binding kinetics of hMPV F variants obtained by biolayer interferometry.

Protein Variants	Assay temperature (°C)	Antibodies					
		MPE8			465		
		koff(1/s)	kon(1/Ms)	KD(M)	koff(1/s)	kon(1/Ms)	KD(M)
M-104	RT	N/A	4.01E+04<br>3.24E+04<br>4.21E+04	N/A	N/A	1.94E+05<br>2.00E+05<br>1.93E+05	N/A
	55	N/A	3.66E+04<br>2.17E+04<br>2.51E+04	N/A	6.60E-05<br>6.85E-05<br>4.25E-05	7.32E+04<br>7.21E+04<br>7.18E+04	9.02E-10<br>9.51E-10<br>5.92E-10
	60	N.B	N.B	N.B	2.29E-04<br>N/A<br>2.62E-04	2.29E+04<br>2.29E+04<br>2.56E+04	1.00E-08<br>N/A<br>1.02E-08
M-305	RT	N.B	N.B	N.B	4.91E-03<br>4.31E-03<br>4.86E-03	1.08E+05<br>1.19E+05<br>1.08E+05	4.52E-08<br>3.61E-08<br>4.51E-08
115-BV	RT	N/A	1.45E+04<br>1.52E+04<br>1.09E+04	N/A	N/A	7.99E+04<br>7.91E+04<br>7.79E+04	N/A
	55	N.B	N.B	N.B	N.B	N.B	N.B
hMPV B2 F (postfusion)	RT	N.B	N.B	N.B	N.B	N.B	N.B
DS-CavEs	RT	N/A	5.12E+04<br>4.92E+04<br>4.93E+04	N/A	2.10E-06<br>1.46E-05<br>N/A	1.74E+05<br>1.92E+05<br>1.90E+05	1.21E-11<br>7.58E-11<br>N/A
	55	N/A	4.49E+04<br>4.02E+04<br>5.08E+04	N/A	1.30E-05<br>1.75E-05<br>1.79E-05	1.51E+05<br>1.47E+05<br>1.59E+05	8.63E-11<br>1.19E-10<br>1.13E-10

	60	0.000151<br>5.60E-05<br>N/A	1.59E+04<br>2.55E+04<br>1.42E+04	9.51E-09<br>2.20E-09<br>N/A	0.000134<br>0.000103<br>0.000181	5.51E+04<br>4.90E+04<br>5.19E+04	2.43E-09<br>2.10E-09<br>3.48E-09
DS-CavEs2	RT	1.33E-05<br>N/A<br>N/A	4.63E+04<br>4.13E+04<br>5.15E+04	2.87E-10<br>N/A<br>N/A	N/A<br>5.16E-06<br>9.32E-06	2.13E+05<br>2.46E+05<br>2.47E+05	N/A<br>2.10E-11<br>3.78E-11
	55	2.89E-05<br>1.33E-05<br>N/A	4.50E+04<br>5.03E+04<br>4.52E+04	6.42E-10<br>2.64E-10<br>N/A	N/A<br>N/A<br>1.79E-05	1.65E+05<br>1.74E+05<br>1.78E+05	N/A<br>N/A<br>1.01E-10
	60	0.000161<br>3.56E-05<br>1.54E-05	4.60E+04<br>4.44E+04<br>4.79E+04	3.49E-09<br>8.00E-10<br>3.22E-10	N/A<br>4.35E-06<br>3.33E-05	1.63E+05<br>1.74E+05<br>1.66E+05	N/A<br>2.50E-11<br>2.00E-10

RT= room temperature
N.B = no binding
N/A = not applicable

Supplementary Table 4. Binding kinetics of SARS-CoV-2 S variants obtained by biolayer interferometry.

**Protein Variants**	**Assay temperature (°C)**	**Prefusion binder**		
		**ACE2**		
		koff(1/s)	kon(1/Ms)	KD(M)
Spk-M	RT	5.69E-04<br>5.54E-04<br>5.82E-04	4.73E+04<br>4.89E+04<br>4.71E+04	1.20E-08<br>1.13E-08<br>1.24E-08
	55	7.12E-04<br>6.06E-04<br>5.41E-04	2.92E+04<br>2.75E+04<br>2.72E+04	2.44E-08<br>2.21E-08<br>1.99E-08
Spk-F	RT	1.07E-03<br>7.14E-04<br>7.86E-04	2.43E+04<br>2.32E+04<br>2.61E+04	4.42E-08<br>3.08E-08<br>3.01E-08
Spk-R	RT	4.00E-04<br>4.61E-04<br>3.43E-04	4.58E+04<br>4.61E+04<br>5.03E+04	8.73E-09<br>1.00E-08<br>6.81E-09
S-2P	RT	N/A<br>2.04E-04<br>N/A	4.26E+04<br>5.60E+04<br>3.93E+04	N/A<br>3.64E-09<br>NA
	55	4.70E-04<br>3.08E-04<br>1.93E-03	2.30E+04<br>1.23E+04<br>7.03E+03	2.04E-08<br>2.51E-08<br>2.74E-07
HexaPro	RT	2.85E-05<br>0.000247<br>9.67E-05	6.53E+04<br>6.00E+04<br>5.86E+04	4.37E-10<br>4.12E-09<br>1.65E-09
	55	1.65E-09<br>0.0013<br>0.00148	2.40E+04<br>2.15E+04<br>2.53E+04	4.75E-08<br>6.05E-08<br>5.86E-08

RT= room temperature
N/A = not applicable

Supplementary Table 5. Data collection and refinement statistics for R-1b and M-104

	**R-1b** (PDB ID 7TN1)	**M-104** (PDB ID 8E15)
**Data collection**		
Space group	P 41 21 2	I 21 3
Cell dimensions		
a, *b*, *c* (Å)	170.5, 170.5, 171.2	178.191, 178.191, 178.191
α, β, γ (°)	90, 90, 90	90, 90, 90
Resolution (Å)	49.3 - 3.1 (3.211 - 3.1)*	47.62 - 2.41 (2.496 - 2.41)*
R_{merge}	0.268 (1.34)	0.03633 (0.921)
$I / \sigma I$	6.3 (1.7)	9.18 (0.78)
Completeness (%)	96.0 (99.47)	99.91 (99.86)
Redundancy	6.4 (7.2)	2.0 (2.0)
**Refinement**		
Resolution (Å)	3.1	2.4
No. reflections	44788 (4518)	36363 (3606)
R_{work} / R_{free}	0.257 (0.297) / 0.315 (0.371)	0.2036 (0.3056) /0.2487 (0.3366)
No. atoms (non-hydrogen)		
Protein	10420	3360
Ligand/ion	42	67
Water	10	11
B-factors		
Protein	71.1	70.11
Ligand/ion	109.5	110.72
Water	30.0	70.94
R.m.s. deviations		
Bond lengths (Å)	0.010	0.009
Bond angles (°)	1.29	1.02

*One crystal was used for each structure.

Values in parentheses are for highest-resolution shell.

Supplementary Table 6. Cryo-EM data collection, refinement, and validation statistics for Spk-M.

	**Spk-M** (EMDB-29035) (PDB 8FEZ)
**Data collection and processing**	
Magnification	22,500
Voltage (kV)	300
Electron exposure (e–/Å^2)	58.24
Defocus range (μm)	-0.8 to -2.6
Pixel size (Å)	1.024
Symmetry imposed	C1
Initial particle images (no.)	1,394,889
Final particle images (no.)	87,514
Map resolution (Å)	3.72
FSC threshold	0.143
Map resolution range (Å)	3.46-9.26
**Refinement**	
Initial model used (PDB code)	6vyb
Model resolution (Å)	3.72
FSC threshold	0.143
Map sharpening *B* factor (Å^2)	46.5
Model composition	
Non-hydrogen atoms	17515
Protein residues	2843
Ligands	0
B factors (Å^2)	
Protein	124.73
Ligand	0
R.m.s. deviations	
Bond lengths (Å)	0.018
Bond angles (°)	1.749
Validation	
MolProbity score	1.75
Clashscore	7.96
Poor rotamers (%)	0.22
Ramachandran plot	
Favored (%)	95.4
Allowed (%)	4.6
Disallowed (%)	0.0

Supplementary Table 7. Immunization doses used during RSV vaccination study.

**Group (Total n)**	**Antigen**	**Prime Vaccination (0 weeks)**	**Boost Vaccination (4 weeks)**
**1** (5)	PBS	–	–
**2** (5)	DS-Cav1	2 µg No adjuvant	2 µg No adjuvant
**3** (5)	DS-Cav1	2 µg + AddaVax	2 µg + AddaVax
**4** (5)	DS-Cav1	0.2 µg No adjuvant	0.2 µg No adjuvant
**5** (5)	DS-Cav1	0.2 µg + AddaVax	0.2 µg + AddaVax
**6** (5)	R-1b	2 µg No adjuvant	2 µg No adjuvant
**7** (5)	R-1b	2 µg + AddaVax	2 µg + AddaVax
**8** (5)	R-1b	0.2 µg No adjuvant	0.2 µg No adjuvant
**9** (5)	R-1b	0.2 µg + AddaVax	0.2 µg + AddaVax
