# Supplementary material for: A general computational design strategy for stabilizing viral class I fusion proteins
Source: Nat Commun. 2024 Feb 13;15:1335. doi: 10.1038/s41467-024-45480-z (PMC10864359; doi:10.1038/s41467-024-45480-z)
Supplement: Supplementary file 3 — Description of Additional Supplementary Files [file 41467_2024_45480_MOESM3_ESM.pdf]

Description of Additional Supplementary Files

File Name: Supplementary Data 1
Description: Computed energetic information of the RSV F protein. The in silico energetic analysis was carried out with the Rosetta software suite and guided the selection of mutations for experimental validation.

File Name: Supplementary Data 2
Description: Computed energetic information of the hMPV F protein. The in silico energetic analysis was carried out with the Rosetta software suite and guided the selection of mutations for experimental validation.

File Name: Supplementary Data 3
Description: Computed energetic information of the SARS-CoV-2 S protein. The in silico energetic analysis was carried out with the Rosetta software suite and guided the selection of mutations for experimental validation.
