# Supplementary material for: A general computational design strategy for stabilizing viral class I fusion proteins
Source: Nat Commun. 2024 Feb 13;15:1335. doi: 10.1038/s41467-024-45480-z (PMC10864359; doi:10.1038/s41467-024-45480-z)
Supplement: Supplementary file 7 — Reporting Summary [file 41467_2024_45480_MOESM7_ESM.pdf]

nature portfolio

Corresponding author(s): Eva-Maria Strauch

Last updated by author(s): 12/04/2023

Reporting Summary

Nature Portfolio wishes to improve the reproducibility of the work that we publish. This form provides structure for consistency and transparency in reporting. For further information on Nature Portfolio policies, see our Editorial Policies and the Editorial Policy Checklist.

Statistics

For all statistical analyses, confirm that the following items are present in the figure legend, table legend, main text, or Methods section.

n/a	Confirmed	
☐	☒	The exact sample size (*n*) for each experimental group/condition, given as a discrete number and unit of measurement
☐	☒	A statement on whether measurements were taken from distinct samples or whether the same sample was measured repeatedly
☒	☐	The statistical test(s) used AND whether they are one- or two-sided<br>*Only common tests should be described solely by name; describe more complex techniques in the Methods section.*
☒	☐	A description of all covariates tested
☒	☐	A description of any assumptions or corrections, such as tests of normality and adjustment for multiple comparisons
☐	☒	A full description of the statistical parameters including central tendency (e.g. means) or other basic estimates (e.g. regression coefficient) AND variation (e.g. standard deviation) or associated estimates of uncertainty (e.g. confidence intervals)
☒	☐	For null hypothesis testing, the test statistic (e.g. *F*, *t*, *r*) with confidence intervals, effect sizes, degrees of freedom and *P* value noted<br>*Give P values as exact values whenever suitable.*
☒	☐	For Bayesian analysis, information on the choice of priors and Markov chain Monte Carlo settings
☒	☐	For hierarchical and complex designs, identification of the appropriate level for tests and full reporting of outcomes
☒	☐	Estimates of effect sizes (e.g. Cohen's *d*, Pearson's *r*), indicating how they were calculated

Our web collection on statistics for biologists contains articles on many of the points above.

Software and code

Policy information about availability of computer code

Data collection	All protein predictions were performed with the Rosetta version:2020.10. post.dev+ 12.master.c 7b9c3e c 7b9c3e4aeb 1 febab2 lld63da2914b 119622e69b.
Data analysis	Custom scripts for data analysis are available on https://github.com/strauchlab/two-state-stabilization. Other software used for analysis: COOT 0.9.8.1, Phenix 1.15, Molprobity (web service version 4.5.1), GatorOne software 1.7.28, cryoSPARC V3.3.2, DeepEM, UCSF Chimera 1.15.

For manuscripts utilizing custom algorithms or software that are central to the research but not yet described in published literature, software must be made available to editors and reviewers. We strongly encourage code deposition in a community repository (e.g. GitHub). See the Nature Portfolio guidelines for submitting code & software for further information.

Data

Policy information about availability of data

All manuscripts must include a data availability statement. This statement should provide the following information, where applicable:

- Accession codes, unique identifiers, or web links for publicly available datasets
- A description of any restrictions on data availability
- For clinical datasets or third party data, please ensure that the statement adheres to our policy

All the databases/datasets used in the study along with appropriately accessible links/accession codes are in the manuscript under the “Data availability” section as well as in this reporting summary. Atomic coordinates of the structures and cryo-EM map reported in this study were deposited in the Protein Data Bank under

accession codes 7TN1 [https://doi.org/10.2210/pdb7TN1/pdb] (R-1b), 8E15 [https://doi.org/10.2210/pdb8E15/pdb] (M-104), and 8FEZ [https://doi.org/10.2210/pdb8FEZ/pdb] (Spk-M), and in the Electron Microscopy Data Bank under accession code EMD-29035 [https://www.ebi.ac.uk/emdb/EMD-29035]. Additional protein structures used in this study are available in the Protein Data Bank under accession codes 5W23 [https://doi.org/10.2210/pdb5W23/pdb], 3RRT [https://doi.org/10.2210/pdb3RRT/pdb], 5C6B [https://doi.org/10.2210/pdb5C6B/pdb], 5WB0 [https://doi.org/10.2210/pdb5WB0/pdb], 5L1X [https://doi.org/10.2210/pdb5L1X/pdb], 6M0J [https://doi.org/10.2210/pdb6M0J/pdb], 6VYB [https://doi.org/10.2210/pdb6VYB/pdb], 6VXX [https://doi.org/10.2210/pdb6VXX/pdb], 6LXT [https://doi.org/10.2210/pdb6LXT/pdb], and 6XRA [https://doi.org/10.2210/pdb6XRA/pdb]. The in silico energetic evaluations generated in this study are provided in Supplementary Data files. Source data are provided as a Source Data file.

Human research participants

Policy information about studies involving human research participants and Sex and Gender in Research.

Reporting on sex and gender	n/a
Population characteristics	n/a
Recruitment	n/a
Ethics oversight	n/a

Note that full information on the approval of the study protocol must also be provided in the manuscript.

Field-specific reporting

Please select the one below that is the best fit for your research. If you are not sure, read the appropriate sections before making your selection.

- [x] Life sciences
- [ ] Behavioural & social sciences
- [ ] Ecological, evolutionary & environmental sciences

For a reference copy of the document with all sections, see nature.com/documents/nr-reporting-summary-flat.pdf

Life sciences study design

All studies must disclose on these points even when the disclosure is negative.

Sample size	We followed the literature and used the minimum number of animals following the IACUC 3R (Replacement, Refine, and Reduction) animal research guideline to observe statistical differences. References: Tang. A. et al. Nat Commun. 2019; 10: 4153. doi: 10.1038/s41467-019-12137-1; Wong T. et al. PLoS One. 2014; 18;9(2):e88764. doi: 10.1371/journal.pone.0088764.<br>For the preparation of computational structures, one hundred relaxed models were generated for each protein, ensuring thorough exploration of the energy landscape. Likewise, the first and second rounds of combinatorial design involved the generation of at least sixty sequences to ensure comprehensive sequence sampling.<br>To validate the computational method, protein expression was conducted for at least three designs from each virus, serving as a proof-of-concept. In the characterization process, a total of ten negative-stain electron micrographs were collected on different areas of each grid, for every protein. Furthermore, cryo-EM analysis involved the collection of a total of 3,257 micrographs.
Data exclusions	No exclusions.
Replication	The initial protein expression was tested once since our objective was to identify which constructs were suitable for further characterization. Subsequently, the selected constructs underwent a second round of expression testing to ensure the reproducibility of results.<br>Mice immunizations were conducted once, testing five biological replicates. This approach is sufficient to observe significant differences, and to minimize the number of mice sacrificed in this study. Repeating the animal experiment is unnecessary.<br>The remaining experiments were performed at least twice and the results were reproducible.
Randomization	Mice were randomly distributed and assigned 5 mice per group in different cages. For protein expression and characterization, the constructs were grouped based on viral family, as adjustments in protein purification and antibody binding assays are necessary based on protein type. Within each viral family, proteins were randomly assigned to transfection and characterization groups.<br>All computational explorations were conducted using random seeds.
Blinding	Within each viral family, the investigators were blinded to group allocation during data collection and analysis.

Reporting for specific materials, systems and methods

We require information from authors about some types of materials, experimental systems and methods used in many studies. Here, indicate whether each material, system or method listed is relevant to your study. If you are not sure if a list item applies to your research, read the appropriate section before selecting a response.

Materials & experimental systems

n/a	Involved in the study	
	x	Antibodies
	x	Eukaryotic cell lines
x		Palaeontology and archaeology
	x	Animals and other organisms
x		Clinical data
x		Dual use research of concern

Methods

n/a	Involved in the study	
x		ChIP-seq
x		Flow cytometry
x		MRI-based neuroimaging

Antibodies

Antibodies used

*D25, Cambridge Biologics, catalog # 01-07-0120.
*AM14, Cambridge Biologics, catalog # 01-07-0119.
*131-2A, Millipore Sigma, catalog # MAB8599.
*Peroxidase-labeled goat anti-mouse IgG, SeraCare, catalog # 5220-0460.
*Anti-RSV polyclonal antibody, EMD Millipore, catalog # AB1128.
*HRP conjugate rabbit anti-goat IgG, Millipore Sigma, catalog # AP106P.
*MPE8, 465, and 101F were produced by Dr. Jarrod Mousa. Reference: Banerjee, A. et al. Structural basis for ultrapotent antibody-mediated neutralization of human metapneumovirus. Proc. Natl. Acad. Sci. U. S. A. 119, 1–9 (2022).

Validation

All monoclonal antibodies were validated in this study by bio-layer interferometry. Binding was assessed against the RSV A2 F, DS-Cav1, or hMPV F 115-BV proteins.
Additionally, the references below can be found on the manufacturer's website statement:
*D25 and AM14: Recombinant human monoclonal antibody recognizing pre-fusion F protein from Respiratory syncytial virus (RSV). Ref: Kwakkenbos, M. J. et al., Generation of stable monoclonal antibody-producing B cell receptor-positive human memory B cells by genetic programming. Nat Med. 2010 Jan;16(1):123-8. doi: 10.1038/nm.2071.
*131-2A: Anti-RSV Antibody, fusion protein, all type A, B strains, clone 131-2A detects level of Respiratory Syncytial Virus and has been published and validated for use in ELISA, flow cytometry, immunofluorescence and western blotting. Ref: Anderson, L. J., Bingham, P. & Hierholzer, J. C. Neutralization of respiratory syncytial virus by individual and mixtures of F and G protein monoclonal antibodies. J Viral. 1988 Nov;62(11):4232-8. doi: 10.1128/JVl.62.ll.4232-4238.1988.

Antibodies provided by Dr. Jarrod J. Mousa were validated in Banerjee, A. et al. Structural basis for ultrapotent antibody-mediated neutralization of human metapneumovirus. Proc. Natl. Acad. Sci. U. S. A. 119, 1–9 (2022).
Additional references validating these antibodies are presented below:
*MPE8, Ref: Corti, D. et al. Cross-neutralization of four paramyxoviruses by a human monoclonal antibody. Nature. 2013 Sep 19;501 (7467):439-43. doi: 10.1038/nature12442.
*465, Ref: Huang, J., Diaz, D. & Mousa, J. J. Antibody recognition of the Pneumovirus fusion protein trimer interface. PloS Pathog. 2020 Oct 9;16(10):e1008942. doi: 10.1371/journal.ppat.1008942.
*101F, Ref: Del Vecchio, A. et al. U.S. patent application 11/261,356.

The anti-RSV polyclonal antibody used in the neutralization assay has been validated by the manufacturer to react against RSV antigens, with applications in indirect immunofluorescence, ELISA, and fusion inhibition.

Eukaryotic cell lines

Policy information about cell lines and Sex and Gender in Research

Cell line source(s)

Freestyle 293-F cells were obtained from Thermo Fisher (Cat. # R79007). Statement from the company: The 293 cell line is a permanent line established from primary embryonal human kidney transformed with sheared human adenovirus type 5 DNA (Graham et al.,1977; Harrison et al., 1977). The FreeStyle 293-F cell line is a variant of the 293 cell line that has been adapted to suspension growth in Freestyle 293 Expression Medium. The 293-F cell line was obtained from Robert Horlick at Pharmacopeia.
The Vero E6 cells used for neutralization assays were obtained from ATCC (Cat. # CRL-1586). Statement from the company: VERO C1008 [Vero 76, clone E6, Vero E6] is a cell line exhibiting epithelial morphology that was isolated from the kidney of an African green monkey. It was cloned by the dilution method into microtiter plates in 1979 by P.J. Price.

Authentication

The cell lines were not authenticated as they were purchased from reputable vendors

Mycoplasma contamination

Cell lines tested negative for mycoplasma contamination

Commonly misidentified lines
(See ICLAC register)

No misidentified cells lines were used in the study.

Animals and other research organisms

Policy information about studies involving animals; ARRIVE guidelines recommended for reporting animal research, and Sex and Gender in Research

Laboratory animals	Six-to-eight-week female BALB/c mice were purchased and housed in individually ventilated Tecniplast SealSafe Plus caging. Mice were housed with a 12-hour photoperiod (light from 7:00 - 19:00 and dark from 19:00 to 7:00) with temperature set at 70°F—72° F and humidity monitored and maintained at 30 - 70%. Food and water were provided ad libitum.
Wild animals	The study did not involve wild animals.
Reporting on sex	Mice are frequently used in RSV infection and vaccination studies, although it is not the ideal animal to replicate the disease observed in humans. RSV infection in mice usually causes lung disease. Female Balb/c mice show the best outcomes with replication of RSV to a high titer in the lungs of BALB/c mice. It's standard and used in several RSV studies.
Field-collected samples	The study did not involve samples collected in the field
Ethics oversight	All animal experiments were performed in accordance with the guidelines and approved protocols by the Institutional Animal Care and Use Committee at the University of Georgia, Athens, USA. The University of Georgia Animal Care and Use program is accredited by AAALAC International (Association for Assessment and Accreditation of Laboratory Animal Care), licensed by the USDA, and maintains an Assurance of Compliance with the Public Health Service.

Note that full information on the approval of the study protocol must also be provided in the manuscript.
